# A Comprehensive Review on Critical Issues and Possible Solutions of Motor Imagery Based Electroencephalography Brain-Computer Interface

**DOI:** 10.3390/s21062173

**Published:** 2021-03-20

**Authors:** Amardeep Singh, Ali Abdul Hussain, Sunil Lal, Hans W. Guesgen

**Affiliations:** School of Fundamental Sciences, Massey University, 4410 Palmerston North, New Zealand; A.abdulhussain@massey.ac.nz (A.A.H.); S.Lal@massey.ac.nz (S.L.); H.W.Guesgen@massey.ac.nz (H.W.G.)

**Keywords:** motor imagery, brain–computer interface (BCI), BCI illiteracy, adaptive BCI, online BCI, asynchronous BCI, BCI calibration, BCI training, electroencephalography (EEG)

## Abstract

Motor imagery (MI) based brain–computer interface (BCI) aims to provide a means of communication through the utilization of neural activity generated due to kinesthetic imagination of limbs. Every year, a significant number of publications that are related to new improvements, challenges, and breakthrough in MI-BCI are made. This paper provides a comprehensive review of the electroencephalogram (EEG) based MI-BCI system. It describes the current state of the art in different stages of the MI-BCI (data acquisition, MI training, preprocessing, feature extraction, channel and feature selection, and classification) pipeline. Although MI-BCI research has been going for many years, this technology is mostly confined to controlled lab environments. We discuss recent developments and critical algorithmic issues in MI-based BCI for commercial deployment.

## 1. Introduction

Numerous people with serious motor disorders are unable to communicate properly if at all. This significantly impacts their quality of life and ability to live independently. In this respect, brain–computer interface (BCI) aims to provide a means of communication. BCIs translate the acquired neural activity into control commands for external devices [1]. Primarily, BCI systems can be cast into various categories that are based on interactions with a user interface and neuroimaging technique applied to capture neural activity. Based on users’ interaction with brain-computer interface, the EEG-BCI system is categorized into synchronous and asynchronous BCI. In the synchronous BCI system, brain activity is generated by the user, which is based on some cue or event taking place in the system at a certain time. This cue helps in differentiating between intentional neural activity for a control signal from unintentional neural activity in the brain [2]. On the other hand, asynchronous BCI works independently of a cue. The asynchronous BCI system also needs to differentiate between neural activity that a user intentionally generates from the unintentional neural activity [3].

Based on neuroimaging techniques, BCI systems fall into invasive and non-invasive categories. In an invasive BCI, neural activity is captured under the skull, thus requiring the surgery to plant the sensors in different parts of the brain. This results in a high-quality signal, is but prone to scar tissue build-up over time, resulting in a loss of signal [4].

Additionally, once the implanted sensors cannot be moved to measures the other parts of the brain [5]. In contrast to this, non-invasive BCI captures brain activity from the surface of the skull. A signal that is acquired through non-invasive technologies has a low signal to noise ratio. Electrocorticography (ECoG) and micro electrodes are some examples of invasive neuroimaging techniques. Electroencephalography (EEG), magnetoencephalography (MEG), functional magnetic resonance imaging (fMRI), and functional near infrared (fNIR) are examples of non-invasive neuroimaging techniques [6]. All of these methods work on different principles and they provide different levels of portability, spatial, and temporal resolution [7]. Among these brain imaging methods, an EEG is widely employed because of its ease of use, safety, high portability, relatively low cost, and, most importantly, high temporal resolution.

Electroencephalography (EEG) is one of the non-invasive and portable neuroimaging techniques that records electrical activity generated due to the synchronized activity of cerebral neurons. Primarily, pyramidal neurons’ activity contributes more to EEG recordings because of their very stable orientated electric field to the cortical surface [6]. This is due to the perpendicular orientation of pyramidal cells with respect to the cortical surface. As a result, the electrical field is projected stably towards the scalp in contrast to the other brain cells whose electrical field is very dispersed and cancels out [7]. The measured EEG signal is due to the complex firing pattern of billions of neurons in the brain. Owing to this pattern, the EEG signal is a combination of various rhythms that reflect certain cognitive states of the individual [7]. These rhythms have different properties, like frequency, amplitude, and shape etcetera. These properties depend upon individual, external stimulus, and the internal state of the individual. Broadly, these rhythms are classified into various categories that are based on their frequency, amplitude, shape, and spatial localization [6]. Furthermore, these rhythms are broadly categorized under six frequency bands: delta band (1–4 Hz), theta band (4–8 Hz), alpha band (8–12 Hz), mu band (8–12 Hz), beta band (13–25 Hz), and gamma band (>25 Hz). EEG control signals can be categorized as evoked and spontaneous. An evoked signal corresponds to neural activity that is generated due to external stimuli. Examples of evoked control signals are steady-state visual-evoked potentials (SSVEP), visual-evoked potentials (VEP), and P300 [4]. On the other hand, a spontaneous control signal is due to voluntarily neural activity without any aid of external stimulus. Slow cortical potentials (SCPs) and sensorimotor rhythms (SMRs) are such control signals [4]. As mentioned above, an evoked control signal requires external stimulus, thus the user needs to focus on presentation to generate neural activity. This continuous focus causes fatigue in users. Nevertheless, much less training is required to generate evoked control signals. Spontaneous control signals offer natural control over neural activity, but they require long training to master self regulation of brain rhythms. To do so, different cognitive tasks are employed to generate spontaneous control signals.

Motor imagery (MI) is one of the most widely used cognitive tasks, which corresponds to sensorimotor rhythms (SMRs) as a control signal. Motor imagery has advantages for the brain–computer interface in both synchronous and asynchronous mode. MI can be defined as the user sending a command to a system through the imagination of a kinesthetic movement of his/her limbs. For example, a user moving a prosthetic arm by imagining his/her left/right hand moving. The imagination of movement creates a similar brain activity to that of an actual movement, which decreases the percentage of power relative to a reference baseline in both the mu and beta frequencies over the sensorimotor cortex; this is known as event related desynchronization (ERD) [8]. Immediately after the user’s imagination task, the user’s brain activity can experience an event-related synchronization (ERS), which is the increase to the percentage of power relative to the reference baseline [8]. Because ERD/ERS are mixed with other brain activity created unintentionally by the user, such as involuntarily muscle movements and eye blinks, the signal to noise ratio (SNR) is low. The algorithm that is designed for MI-BCI must be able to differentiate between MI activity for control signal from other involuntary activity. In doing so, the MI-BCI pipeline consists of many stages, like data acquisition, preprocessing, feature extraction, and classification. Therefore, the objective of this manuscript is to review the MI based BCI system with regards to algorithms that were utilized at different stages of MI-BCI pipeline. This brief survey is structured under an architectural framework that helps in mapping the literature to each component of the MI-BCI pipeline. In doing so, this article identifies critical research gaps that warrant further exploration along with current developments to mitigate these issues.

**Figure 1:**
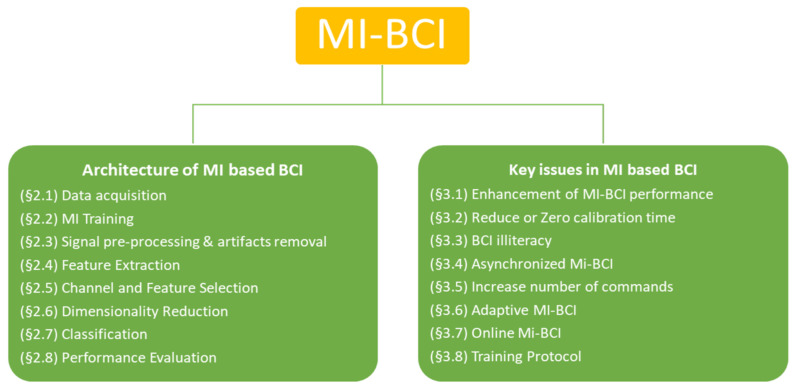
Breakdown of the article.

Figure 1 breaks down the contents of the entire article. This review article is divided into two parts. The first part of this article introduces the Architecture of MI based BCI. More specifically, how the EEG signal is captured from the brain is described under Section 2.1. In Section 2.2, we discuss how during the calibration phase the user acquires skills to modulate brain waves into control commands. The signal pre-processing subsection explains how unwanted artifacts are removed from the EEG signal to improve the signal to noise ratio. Section 2.4 discusses different approaches to extract information that is related to a motor imagery event in terms of features that are finally classified into control commands. Subsection on Section 2.5 and Section 2.6 deals with issues related to finding optimal channels or features and reducing dimensionality of feature space in order to improve BCI performance. Section 2.7 provides details of how features are classified into control commands. Lastly, Section 2.8 covers how to evaluate the performance of BCI. The last part of this article discusses the key issues that need further exploration along with the current state of the art that address these research challenges.

## 2. Architecture of MI Based BCI

**Figure 2:**
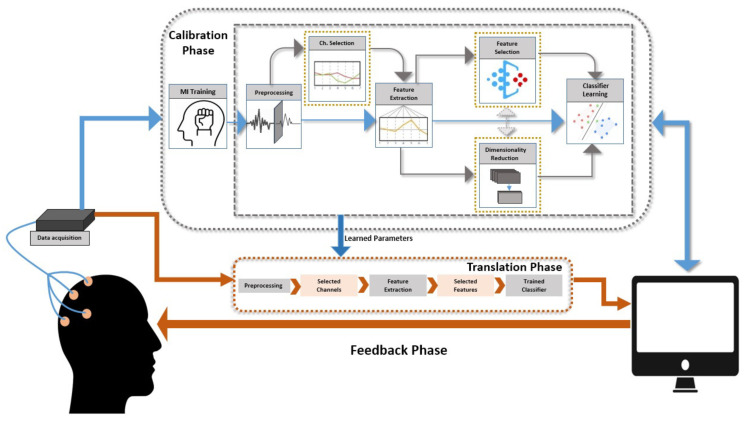
Block diagram showing the typical structure of MI-based brain–computer interface (BCI).

We present a framework of MI-BCI pipeline encompassing all of the components that are responsible for its working in Figure 2. In short, MI-BCI works in calibration and online mode, respectively. During calibration mode, the user learns voluntary ERD/ERS regulation in the EEG signal and BCI learns ERD/ERS mapping through temporal, spectral, and spatial characteristics of the user’s EEG signal. In online mode, the user’s characteristics are translated into a control signal for external application and feedback is given to the user. In framework, optional steps that are enclosed in yellow box, such as channel selection, feature selection, and dimensionality reduction. This framework is also helpful in mapping the literature to different components of the MI-BCI pipeline in order to understand the current research gaps.

### 2.1. Data Acquisition

The signal acquisition unit is represented by electrodes whether they are invasive or non-invasive. In the non-invasive approach, electrodes are usually connected with the skin via conductive gel to create a stable electrical connection for a good signal. The combination of conductive gel and electrode attenuate the transmission of low frequencies, but take a very long time to setup. Another alternative is dry electrodes, which make direct contact with skin without conductive gel. Dry electrodes are easy and faster to apply, but are more prone to motion artifacts [5]. EEG signals are usually acquired under unipolar and bipolar modes. In unipolar mode, a potential difference between all the electrodes with respect to one reference are acquired. Each electrode-reference pair form one EEG channel. On the contrary, in bipolar mode, the potential difference between two specified electrodes are acquired and each pair make a EEG channel [9]. To standardize positions and naming, electrodes are placed on the scalp under international 10–20 standard. This helps in reliable data collection and consistency among different BCI sessions [10].

**Figure 3:**
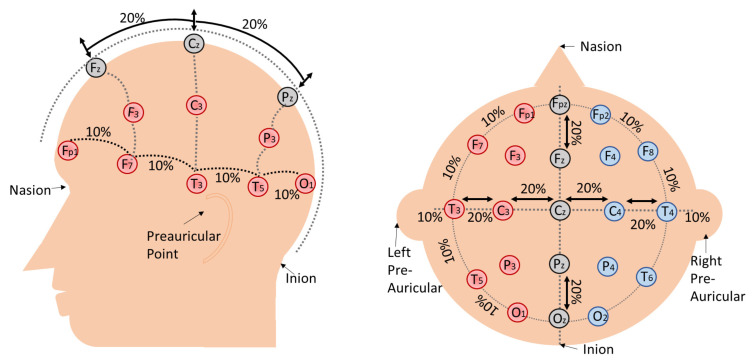
The international 10–20 standard electrode position system.The left image presents a left side view of the head with electrode positions, and the right image shows a top view of the head.

Figure 3 shows the international 10–20 electrodes’ placement scheme from the side and top view of the head. Once the potential difference has been identified by the EEG electrodes, it is amplified and digitized in order to store it in a computer. This process can be expressed as taking one sample (discrete snapshots) of the continuous cognitive activity. This discrete snapshot (sample) depends on the sampling rate of the acquisition device. For example, an EEG acquisition device with a sampling rate of 256 Hz can take 256 samples per second. High sampling rates and more EEG channels are used to increase the temporal and spatial resolutions of an EEG acquisition device.

### 2.2. MI Training

During calibration phase, the user learns how to modulate EEG signals with MI task pattern. Just as with any skill, MI training helps in acquiring the ability to produce a distinct and stable EEG pattern while performing the different MI tasks [11]. The Graz training paradigm is the standard training approach for motor imagery [8,11]. The Graz approach is based on machine learning, where the system adapts with the user’s EEG pattern. During this training paradigm, the user is instructed through a cue to perform a motor imagery task, such as left and right-hand imagination. EEG signals that are collected during different imagination tasks are used to train the system differentiate between the MI-tasks from the EEG pattern. Once the system is trained, users are instructed to perform MI tasks, but this time feedback is provided to the user. This process is repeated multiple times over different sessions. Each session has further multiple runs of the Graz training protocol.

**Figure 4:**
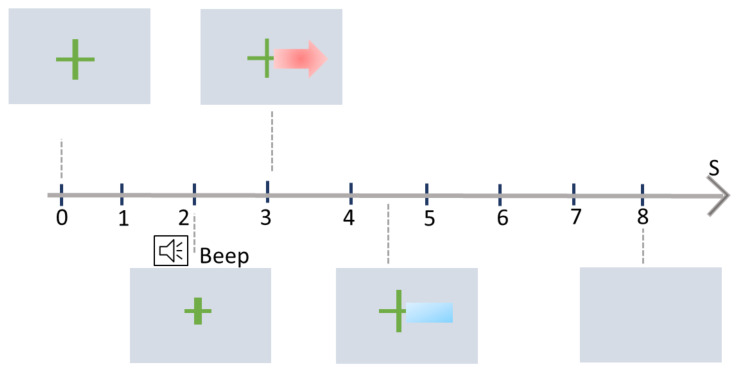
An illustration of one trial’s timing in the Graz protocol [11].

The trial time vary depending on scenario. Typically, one trial of graz training protocol lasts eight seconds, as illustrated in Figure 4. At the outset of each MI trail, which is t=0 s, a fixation cross is displayed to instruct the user that the trial has started. After a two-second break (t=2 s), a beep is used to prepare the user for the upcoming MI task. This 2 s break acts as a baseline period to see the MI task pattern in the EEG signal in the upcoming MI task at t=3 s. After three seconds, an arrow appears on the screen indicating the MI task. For example, the arrow in the right direction means right hand motor imagery. No feedback is provided during the initial training phase. After the system is calibrated, feedback is provided for four seconds. The direction of the feedback bar shows recognition of the MI pattern by system and the length of the bar represents confidence of the system in its recognition of the MI class pattern.

Various other extensions of the Graz paradigm is proposed in the literature, mostly focusing on providing alternative MI instructions and feedback from the system. For example, the bar feedback is replaced by auditory [12] and tactile [13] feedback to reduce the workload on the visual channel. Similarly, virtual reality based games and environments are explored to provide MI instructions and feedback for training [14,15].

### 2.3. Signal Pre-Processing and Artifacts Removal

Artifacts are nothing but unwanted activities during signal acquisition. They are comprised of an incorrect collection of signal or signals acquired from areas other than the cerebral origin of the scalp area. Generally, artifacts are classified into two major categories, termed as endogenous and exogenous artifacts. Endogenous artifacts are generated from the human body excluding the brain, and extra-physiologic artifacts are generated from external sources (i.e., sources from outside the human body) [7]. Some of the common endogenous and exogenous artifacts that accrue during EEG signal acquisition are bad electrode position, poor ground electrode, obstructions to electrode path (e.g., hair), eye blinks, electrode impedance, electromagnetic noise, equipment problem, power line interference, ocular artifacts, cardiac artifacts, and muscle disturbances [16]. The signal pre-processing block is responsible for the removal of such exogenous and endogenous artifacts from the EEG signal. MI-BCI systems mainly rely on a temporal and spatial filtering approach.

Temporal filtering is the most commonly used pre-processing approach for EEG signals. Temporal filters are usually low pass or band pass filters that are used to restrict signals in the frequency band where neurophysiological information relevant to the cognitive task lies. For MI, this usually means a Butterworth or Chebyshev bandpass filter of 8–30 Hz frequency. This bandpass filter keeps both the mu and beta frequency bands as they are known to be associated with motor-related tasks [8]. However, MI task-related information is also present in the spatial domain. Similar to temporal filters, spatial filters extract the necessary spatial information associated with a motor-related task embedded in EEG signals. A common average reference (CAR) is a spatial filter that removes the common components from all channels, leaving channels with only channel specific signals [17]. This is done by removing the mean of all *k* channels from each channel $x_{i}$:

(1) $$\begin{array}{c} x_{i}^{\mathrm{CAR}}=x_{i}-mean\left(x_{i}\right). \end{array}$$

CAR benefits from being a very computationally cheap approach. An updated version of CAR is the Laplacian spatial filter. The Laplacian spatial filter aims to remove the common components of neighboring signals, which increases the difference between channels [18]. The Laplacian spatial filter is calculated through the following equation:

(2) $$\begin{array}{cc} V_{i}^{LAP} & =V_{i}^{ER}-\sum_{j\in S_{i}}g_{ij}V_{j}^{ER} \end{array}$$

(3) $$\begin{array}{cc} g_{ij} & =\frac{d_{ij}}{\sum_{j\in S_{i}}d_{ij}} \end{array}$$

where $V_{i}^{LAP}$ is the ith channel that is filtered by the Laplacian method, $V_{i}^{ER}$ is the potential difference between ith electrode and reference electrode, $S_{i}$ is the set of neighboring electrodes to the ith electrode, and $d_{ij}$ is the Euclidean distance between ith and jth electrode [18].

### 2.4. Feature Extraction

Measuring motor imagery through an EEG leads to a large amount of data due to high sampling rate and electrodes. In order to achieve the best possible performance, it is necessary to work with small values that are capable of discriminating MI task activity from unintentional neural activity. These small values are called “features” and the process to achieve these values is called “feature extraction”. Formally, feature extraction is the mapping of preprocessed large EEG data into a feature space. This feature space should contain all of the necessary discrimination information for a classifier to do its job. For MI-BCI, the feature extraction methods can be divided into six categories: (a) time domain methods that exploit temporal information embedded in the EEG signal; (b) spectral methods extract information that is embedded in the frequency domain of EEG signals; (c) time-frequency methods works together on information in the time and frequency domain; (d) spatial methods extract spatial information from EEG signals coming from multiple electrodes; (e) spatio-temporal methods works together with spatial and temporal information to extract features; (f) spatio-spectral methods use spatial and spectral information of the multivariate EEG signals for feature extraction; and, (e) Riemannian Manifold methods, which are essentially a sub category of spatio-temporal methods that exploits manifold properties of EEG data for feature extraction. Table 1 summarizes all of the feature extraction methods discussed in the following subsections.

#### 2.4.1. Time Domain Methods

An EEG is a non-stationary signal whose amplitude, phase, and frequency changes with SMR modulations. Time domain methods investigate how the SMR modulation changes as a function of time [35]. Time domain methods work on each channel individually and extract temporal information related to the task. The extracted features from different channels are fused together to make a feature set for a single MI trial. In MI-BCI literature, statistical features, like mean, root mean square(RMS),integrated EEG (power of signal), standard deviation, variance, skewness, and kurtosis, are vastly employed to classify MI tasks [19,20]. Other alternative time domain methods that are based on variance of signal are Hjorth parameters. A Hjorth parameter measures power (activity), mean frequency (mobility) and change in frequency (complexity) of EEG signal [21]. Similarly, fractal dimension (FD) is non-linear method that measures EEG signals complexity [22]. Auto-regressive (AR) modeling of the EEG signal is another typical time domain approach. The AR models signal from each channel as a weighted combination of its previous samples and AR coefficients are used as features. An extension of AR modelling is adaptive auto regressive modelling (AAR) and it is also used for MI-BCI studies. Unlike AR, the coefficients in AAR are not constant and, in fact, varies with time [21]. Information theory-based features, like entropy, are also used in time domain to quantify complexity of the EEG signal [25]. Temporal domain entropy works with amplitude of EEG signal [26].

Another way of extracting temporal information is to represent the signal in terms of peaks (local maximum) and valley (local minimum) [23]. In this peak-valley representation, various features points are extracted between neighbouring peak and valley points. Using the peak-valley model, Yilmaz et al. [24] approximated EEG signal in 2D vector that contains cosine angle between transition points (peak or valley) and normalized the ratio of Euclidean distance in a left/right transition (peak or valley) points. In the same vein, Mendoza et al. [27] proposed a quaternion based signal analysis that represents a multi-channel EEG signal in terms of their orientation and rotation then obtained statistical features for classification. Recently, EEG signal analysis based on graph theory and functional connectivity (FC) is employed in MI-BCI [36]. These methods take advantage of the functional communication between the brain regions during cognitive task like MI. In graph based methods, the EEG data are represented through graph adjacency matrices that correspond to temporal correlations (correlation approaches used like Pearson or Correntropy) between different brain regions (electrodes). Features are extracted from this graph in terms of the graph node’s importance, such as centrality measure [17].

The advent of data driven approaches, like deep learning, has largely alleviated the need for hand crafted features. In these approaches, a raw or preprocessed EEG signal is passed through different convolution and pooling layers to extract temporal information [37]. In the same vein, Lawhern et al. [38] proposed EEGNET deep learning architecture that works with raw EEG signals. It starts with a temporal convolution layer to learn the frequency filters (equivalent to preprocessing), another depth-wise convolution layer is used to learn frequency-specific spatial filters. Lastly, a combination of a depth-wise convolution along with point wise convolution are used to fuse features coming form previous layers for classification. Instead of using a raw or preprocessed signal, another approach is for the signal to be approximated and then passed to a deep neural network model. A one dimension-aggregate approximation (1d-AX) is one way of achieving this [39]. 1d-AX takes a signal from each channel in a single trial, normalizes it, and applies linear regression. These regression results are passed as features to the neural network.

#### 2.4.2. Spectral Domain Methods

Spectral methods extract information from EEG signals in the frequency domain. Similar to the temporal method, statistical methods are also applied in the frequency domain. Samuel et al. [19] used statistical methods in both time and frequency domain to decode motor imagery. The most used spectral method is the power (energy) of EEG signals in specific frequency band. Usually, spectral power is calculated in mu (μ), beta (β), theta (θ), and delta (δ) frequency bands. This is done by decomposing the EEG signal into its frequency components at the chosen frequency band while using Fast Fourier Transform (FFT) [28,40]. The other frequency domain based method is Power Spectral Density (PSD). PSD is the measure of how the power of a signal is distributed over frequency. There are multiple methods of estimating it, such as Welch’s averaged modified periodogram [41], Yule–Walker equation [42], or Lomb–Scargle periodogram [43]. Spectral entropy is another spectral feature that relies on PSD to quantify information in the signal [44].

#### 2.4.3. Time-Frequency Methods

Time-frequency (t-f) methods works simultaneously in both temporal and spectral domains to extract information in signal. One of the approaches used in t-f domain is the short Term Fourier Transform (STFT), which segments the signal into overlapping time frames on which FFT is applied by the fixed window function [28]. Another way to generate t-f spectra is through a wavelet transform [29], which decomposes the signal into wavelets (finite harmonic functions (sin/cos)). This captures the characteristics in the joint time-frequency domain. Another similar method in the t-f domain is empirical mode decomposition (EMD) [30]. However, instead of decomposing the signal into wavelets, it decomposes a signal x(t) into simple oscillatory functions, called Intrinsic Mode Functions (IMFs) [45]. IMFs are a orthogonal representation of signals, such that first IMF captures a higher frequency and subsequent IMFs capture lower frequencies in EEG signals. Table 1 sums up all the t-f methods.

#### 2.4.4. Spatial Domain Methods

Unlike temporal methods that work with only one channel at a time, spatial domain methods work with multiple channels. Spatial methods try to extract features by finding a combination of channels. This can be achieved while using blind source separation (BSS) [46]. BSS assumes that every single channel is the sum of clean EEG signals and several artifacts. Mathematically, this looks like the following:

$$\begin{array}{c} x(t)=As(t) \end{array}$$

where x(t) is the channels, s(t) is the sources, and *A* is mixing matrix. They aim to find a matrix *B* that reverse the channels back into their original sources:

$$\begin{array}{c} s^{\prime }\left(t\right)=Bx\left(t\right). \end{array}$$

Examples of a BSS algorithms are Cortical current density (CCD) [32] and independent component analysis (ICA) [33]. BSS methods are unsupervised; thus, relations between the classes and features are unknown. However, there exist a supervised method that extract features based on class information, and one of such method is Common Spatial Pattern (CSP). CSP is based on the simultaneous diagonalization of two classes of EEG of their two respective estimated covariance matrices. CSP aims at learning a projection matrix *W* (spatial filters) that maximizes the variance of signal from one class while minimizing the variance from the other class [31]. This is mathematically represented as:

$$\begin{array}{c} J\left(w\right)=\frac{w^{T}C_{1}w}{w^{T}C_{2}w} \end{array}$$

where $C_{1},C_{2}$ represent the estimated co-variance matrix of each MI class. The above equation can be solved while using the Lagrange multiplier method. CSP is known to be highly sensitive to noise and performs poorly under small sample settings, thus a regularized version has been developed [31]. There are two ways to regularize the CSP algorithm (also known as regularized CSP), either by penalizing its objective function J(w), or regularizing its inputs (covariance matrices) [31]. One can regularize the objective function by adding a penalty term to the denominator:

$$\begin{array}{c} J\left(w\right)=\frac{w^{T}C_{1}w}{w^{T}C_{2}w+\alpha P\left(w\right)} \end{array}$$

where P(.) is a penalty function, and α is a constant that is determined by the user (α=0 for CSP) [31]. While CSP inputs can be regularized by:

$$\begin{array}{cc} \tilde{C_{c}} & =\left(1-\gamma \right)\bar{C_{c}}+\gamma I\\ \bar{C_{c}} & =\left(1-\beta \right)s_{t}C_{c}+\beta G_{c} \end{array}$$

where $s_{t}$ is a scalar and $G_{c}$ is a “generic” covariance matrix [31]. CSP performance becomes limited when the EEG signal is not filtered in the frequency range appropriate to subject. To address this issue, the filter bank CSP (FBCSP) algorithm is proposed that passes the signal though multiple temporal filters and CSP energy features are computed from each band [47]. Finally, CSP features from sub-bands are fused together for classification. This results in a large number of features, which limits the performance. To address this alternative method, sub-band common spatial pattern (SBCSP) is proposed, which employs linear discriminant analysis (LDA) to reduce the dimensionality. Finding multiple sub-bands to compute CSP energy features increases the computational cost. To solve this, discriminant filter bank CSP (DFBCSP) is proposed, which utilizes the fisher ratio (FR) to select most discriminant sub-bands from multiple overlapping sub-bands [48].

#### 2.4.5. Spatio-Temporal and Spatio-Spectral Methods

Spatio-temporal methods are algorithms that manipulate both time and space (channels) domains. The main spatio-temporal methods that are used in past MI-BCI studies are Riemannian Manifold-based methods (discussed in the next section). Other spatio-temporal methods are usually based on deep learning. Echeverri et al. [46] proposed one such approach, which uses the BSS algorithm to separate the input signal x(t) from a single channel into an equal number of estimated source signals $\hat{s}\left(t\right)$. These source signals are sorted, based on a correlation between their spectral components. Finally, continious wavelet transform is applied to sorted source signals to achieve t-f spectra images that are further subjected to a convolution neural network (CNN) for classification. In the same vein, Li et. al. [49] proposed an end-to-end EEG decoding framework that extracts the spatial and temporal features from raw EEG signals. In a similar manner, Yang et al. [50] proposed a combination long short-term memory network (LSTM) and convolutional neural network that concurrently learns temporal and spectral correlations from a raw EEG signal. In addition, they used discrete wavelet transformation decomposition to extract information in the spectral domain for classification of the MI task.

Like spatio-temporal methods, spatio-spectral methods extract information from spectral and spatial domains. Temporal and spatial filters are usually learned in sequential (linear) order, whereas, if they are learned simultaneously, a unified framework will be able to extract information from spatial and spectral domains. For instance, Wu et al. [51] employed a statistical learning theory to learn most discriminating temporal and spectral filters simultaneously. In the same vein, Suk and Lee [52] used a particle-filter algorithm and mutual information between feature vectors and class labels to learn spatio-spectral filters in a unified framework. Similarly, Zhang et al. [53] proposed a deep 3-D CNN network that was based on AlexNet that learns spatial and spectral EEG representation. Likewise, Bang et al. [54] proposed a method that generates 3D input feature matrix for the 3-D CNN network by stacking multiple-band spatio-spectral feature maps from multivariate EEG signal.

#### 2.4.6. Riemannian Geometry Based Methods

**Figure 5:**
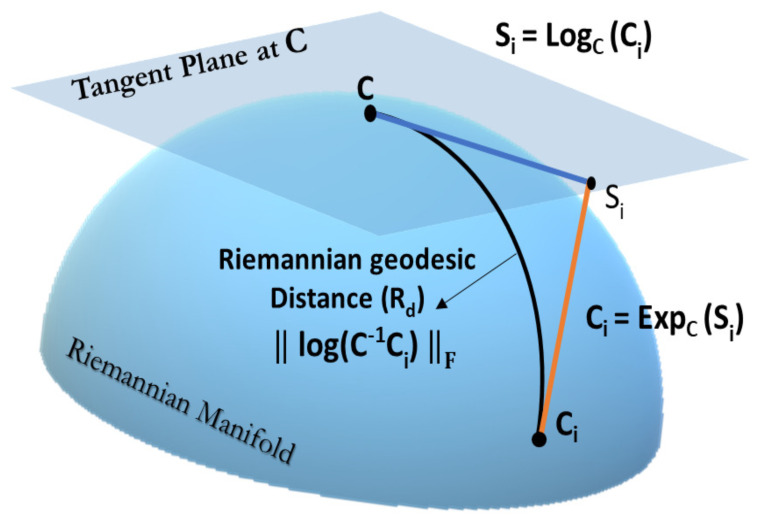
An image of the Riemannian Manifold displaying an example of a geodesic (the shortest distance between two Riemannian points), tangent space, and tangent mapping.

Sample covariance matrices (SCM) calculated from EEG signals are widely used in BCI algorithms. SCM lie in the space of symmetric positive definite (SPD) matrices $P\left(n\right)=\left\{P=P^{T},u^{T}Pu>0,\forall u\in R^{n}\right\}$ which forms a Riemannian Manifold [34]. Unlike the Euclidean space, the distance in the Riemannian manifold are curves, as shown in Figure 5. These curves can be measured while using Affine invariant Riemannian metric (AIRM) [55]: Let $X,Y\in S_{+}^{n}$ be two SPD matrices. Then, the AIRM is given as

$$\begin{array}{c} \delta_{r}^{2}\left(X,Y\right)=∥\mathrm{Log}\left(X^{-1/2}YX^{-1/2}\right){∥}_{F}^{2}={∥\mathrm{Log}\left(X^{-1}Y\right)∥}_{F}^{2}. \end{array}$$

Thus, methods in the Euclidean space can not be directly applied to SCMs. One way of using Euclidean methods to deal with SCMs is to project the SCM into a tangent space (see Figure 5). Because the Riemannian manifold (in fact any manifold) locally looks Euclidean, a reference point $P_{ref}$ for the mapping that is as close as possible to all data points must be chosen. This reference point is usually a Riemannian mean $P_{ref}=\sigma \left(P_{i}\right)$.

### 2.5. Channel and Feature Selection

**Figure 6:**
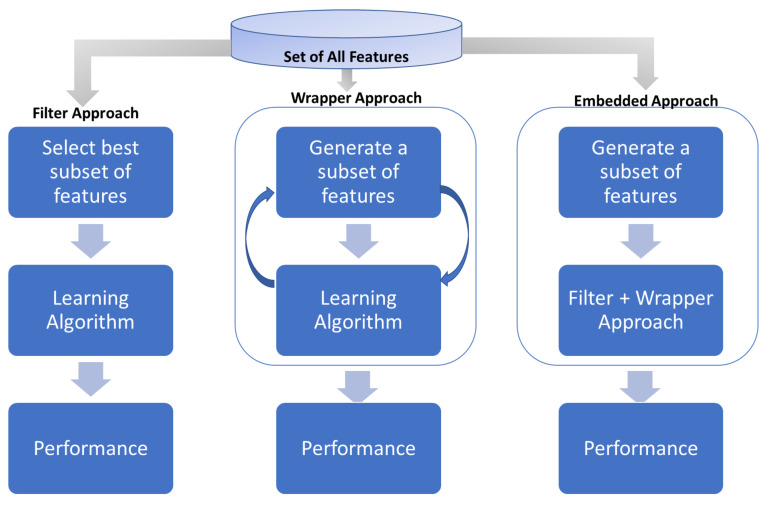
Flow diagram of different feature selection approaches.

EEG data are usually recorded through a large number of locations across the scalp. This provides a higher spatial resolution and benefits in identifying optimal locations (channels) that are relevant to BCI application or task. Here channel selections techniques significantly contribute to identify optimal channels for particular BCI application. Finding optimal channels not only reduces the computational cost of the system, but also reduces the subject’s inconvenience due to the large number of channels. Thus, the main objective of channel selection methods is to identify optimal channels for the BCI task for improving the classification accuracy and reducing computation time in BCI. The channels’ selection problem is similar to that of feature selection, where a subset of important features are selected from a vast number of features. Therefore, channel selection techniques are derived from feature selection algorithm. Once the channels are selected, we still need to extract features for classification of the BCI task. We are sometimes even required to use the feature selection algorithm on selected channels to improve the performance of the system. Feature or channel selection algorithms have many stages. Firstly, a candidate subset of features or channels are generated from an original set for evaluation purposes. This candidate subset is evaluated with respect to some selection criterion. This process is repeated for each candidate subset until a stopping criterion is reached. The selection criteria are what differentiates feature selection approaches. There are two stand-alone feature selection approaches filter approach and wrapper approach. A combination of both is sometimes used to make hybrid approaches also known as embedded approach. The embedded method exploits the strengths of both filter and wrapper approaches by combining them in feature selection process. Figure 6 shows a flow diagram of the above-mentioned feature selection techniques.

#### 2.5.1. Filter Approach

Filter methods starts with all of the features and selects the best subset of features based on some selection criteria. This selection criteria are usually based on characteristics, such as information gain, consistency, dependency, correlation, and distance measures [56]. The advantage of filter methods are their low computational cost and selection of features is independent of the learning algorithm (classifier). Some of the most widely employed filter methods are correlation criteria and mutual information. Correlation detects the linear dependence between variables $x_{i}$ (features) and target *Y* (MI task classes). It is defined as:

$$R\left(i\right)=\frac{cov(x_{i},Y)}{\sqrt{var\left(x_{i}\right)var\left(Y\right)}}$$

where cov() is the covariance and var() the variance. Mutual information (I) and its variant are widely used feature selection filter approaches in the MI-BCI literature. Mutual Information [57] $I(c_{i};f)$ is the measure of the mutual dependence and uncertainty between two random variables: the features *f* and the classes $c_{i}$. This is measured by subtracting the uncertainty of the class $H(c_{i})$ (that is also called initial uncertainty) from the uncertainty of the class given the features $H(c_{i}|f)$:

$$\begin{array}{c} I\left(c_{i};f\right)=H\left(c_{i}\right)-H\left(c_{i}|f\right) \end{array}$$

Class uncertainty $H(c_{i})$ and class uncertainty, both given the features $H(c_{i}|f)$, can be measured using Shannon’s information theory entropy:

$$\begin{array}{cc} H(c_{i}) & =-\sum_{i=1}^{2}P\left(c_{i}\right)\log P\left(c_{i}\right)\\ H(c_{i}|f) & =-\sum_{f=1}^{N_{f}}P\left(f\right)\left(\sum_{i=1}^{2}P\left(c_{i}|f\right)\log P\left(c_{i}|f\right)\right) \end{array}$$

where $P(c_{i})$ is the probability density function of class $c_{i}$, and $P(c_{i}|f)$ is the conditional probability density function. When mutual Information is equal to zero $I(c_{i}|f)=0$, the class $c_{i}$ and the feature *f* are independent, and, as MI gets higher, the more relevant feature *f* to class $c_{i}$. Thus, MI can be used to select the features by relevance.

Similarly, *t*-test [58] measures the relevance of a feature to a class. It achieves this by examining mean $\mu_{i,j}$ and $\sigma_{i,j}$ variance of a feature $f_{j}$ between class i={1,2} through the following equation:

$$\begin{array}{c} T\left(f_{j}\right)=\frac{|\mu_{1,j}-\mu_{2,j}|}{\sqrt{\frac{\sigma_{1,j}^{2}}{n_{1}}+\frac{\sigma_{2,j}^{2}}{n_{2}}}} \end{array}$$

where $n_{i}$ (n1 and n2) is the number of trials in class i={1,2}. This is then used to select a subset using the highest scoring features. Correlation based feature selection (CFS) [59] evaluates subsets of features based on the hypothesis that a good subset is the one that contains features that are highly correlated with the output classes and not correlated between them. This is computed using heuristic metric $Metric_{S}$ that divides the productiveness of *k* feature subset *S* by the redundancy that exists in the *k* features that compose the subset *S*:

$$\begin{array}{c} Metric_{S}=\frac{k\bar{r_{cf}}}{\sqrt{k+k\left(k-1\right)\bar{r_{ff}}}} \end{array}$$

where $\bar{r_{cf}}$ is the mean of the class-feature correlation, $\bar{r_{ff}}$ is the mean of the inter-feature correlation.

*F-score* [60] is another feature selection approach that quantifies the discriminative ability of variables (features) based on the following equation:

$$F-score_{i}=\frac{\sum_{k=1}^{c}{\left(\bar{x_{i}^{k}}-\bar{x_{i}}\right)}^{2}}{\sum_{k=1}^{c}\left[\frac{1}{N_{i}^{k}}-1\sum_{j=1}^{N_{i}^{k}}\left(x_{ij}^{k}-\bar{x_{i}^{k}}\right)\right]}\left(i=1,2\ldots ,n\right)$$

where *c* is the number of classes, *n* is the number of features, $N_{i}^{k}$ number of samples of feature *i* in class *k*, and $x_{ij}^{k}$ is the jth training sample for feature *i* in class *k*. Features are ranked based on *F-score*, such that a higher *F-score* value corresponds to most discriminative feature.

#### 2.5.2. Wrapper Approach

Wrapper approaches select a subset of features, present them as input to a classifier for training, observe the resulting performance, and stop the search according to a stopping criterion or propose a new subset if the criterion is not satisfied [56]. Algorithms that fall under the wrapper approach are mainly searching and evolutionary algorithms. Searching algorithms start with an empty set and add features (remove features) until a maximum possible performance from the learning algorithm is reached. Usually, a searching algorithm’s stopping criteria is until the number of features reaches a maximum size that is specified for the subset. On the other hand, evolutionary algorithms, such as particle swarm optimization (PSO) [61], differential evolution (DE) [62,63], and artificial bee colony (ABC) [64,65], find an optimal feature subset by maximizing fitness function’s performance. Wrapper methods find a more optimal feature subset when compared to filter methods, but the computational cost is very high, thus not being suitable for very large data-sets.

### 2.6. Dimensionality Reduction

In contrast to feature selection techniques, dimensionality reduction methods tends to reduce the number of features in data, but they do so by creating new combinations (transformation) of features, whereas feature selection methods, achieve this by including and excluding features from the original feature set. Mathematically, dimensionality reduction can be defined as the transformation of high dimensional data ($X\in R^{D}$) into a lower dimensional data ($Z\in R^{d}$), where d<<D. The dimensional reduction techniques can be categorized based on their objective function [66]. Those that are based on optimizing an convex (no local optima) objective function are convex techniques where as techniques whose optimization function may have local optima are non-convex techniques. Furthermore, these techniques can be linear or non-linear based on the transform function used to map high dimensional to low dimension. The most used linear-convex technique is the Principal Component Analysis (PCA), which transforms data in a direction that maximizes the variance in the data set [67,68]. In a similar vein, Linear Discriminant Analysis (LDA) [69] is a linear dimensional reduction technique that finds a subspace that maximizes the distance between multiple classes. To do so, it uses class labels whereas PCA is an unsupervised technique. Independent Component Analysis (ICA) is another linear method that is found in EEG-BCI literature for dimensionality reduction, which works on the principle that the EEG signal is a linear mixture of various sources and all sources are independent of each other [70]. To address the non-linearity in a data-points structure, PCA can be extended by embedding it with a kernel function (KPCA) [70]. KPCA first transforms the data from the original space into kernel space using non-linear kernel transformation function, and then PCA is applied in kernel space. Likewise, Multilayer Autoencoders (AE) is an unsupervised, non-convex. and non-linear technique fpr reducing the dimensionality of data [66]. AE [71] takes the original data and reconstructs into lower dimensional output using the neural network. The drawback of the above discussed methods is that they do not consider the geometry of data prior to transformation. Thus, manifold learning for dimensionality reduction has recently gained more attention in MI-BCI research.

Manifold learning-based methods recover the original domain structure in reduced dimensional structure of data. Generally, these methods are non-linear and divided into global and local categories based on data matrix used for mapping high-dimensional to low-dimensional. Global methods used full EEG data covariance matrix and aim to retain global structure, and do not take the distribution of neighbouring points into account [72]. Isometric feature mapping (Isomap) [73,74] and diffusion maps [73,75] are some of these global methods. In order to preserve global structure of manifold, isomap and diffusion maps aim to preserve pairwise geodesic distance and diffusion distance between data-points, respectively. In contrast, local methods use a sparse matrix to solve eigenproblems, and their goal is to retain the local structure of the data. Locally, Linear Embedding [76,77], Laplacian eigenmaps [74], and local tangent space alignment (LTSA) [78] are some of these local methods. LLE assume manifold is linear locally and thus reconstruct data point from linear combination of its neighbouring points. Similar to LLE, Laplacian Eigenmaps [74] preserve the local structure by computing low-dimensional subspace, in which the pairwise distance between a datapoint and its neighbours is minimal. Similarly, the LTSA [78] maps datapoints in high dimensional manifold to its local tangent space and there reconstruct the low dimensional representation of the manifold. All of the above methods are designed for a general manifold, thus approximating the geodesic distance without information of the specific manifold. The EEG covariance matrix lies in Riemannian manifold; therefore, more methods focused on dimensionality reduction are developed.

When considering the space of EEG matrices in Riemannian manifold, Xie et al. [78] proposed bilinear sub-manifold learning (BSML) that preserve the pairwise Riemannian geodesic distance between the data points instead of approximating the geodesic distance. Likewise, Horev et al. [55] extended PCA in Riemannian manifold by finding a matrix $W\in R^{n\times p}$ that maps the data from the current Riemannian space to a lower dimension Riemannian space while maximising variance. Along the same context, Davoudi et al. [79] proposed a nonlinear dimensionality reduction methods that preserves the distances to the local mean (DPLM) and takes the geometry of the symmetric positive definite manifold into account. Tanaka et al. [80] proposed creating a graph that contains the location electrodes and their respective signals, and later applies the graph Fourier transform (GFT) to reduce the dimensions.

### 2.7. Classification

Classification is the mapping of the feature space $\left(Z\in R^{d}\right)$ into the target space (y∈TargetSpace). This mapping is usually created by three things: a mapping function f∈FunctionSpace, an objective function J(w), and a minimization/maximization algorithm (iterative or by direct calculation). Each of these has a role in the classifications process. The mapping function *f* determines both the space at that is being worked on and the approximation abilities of the classifier, whereas the objective function J(w) describes the problem that the classifier aims to solve. Finally, the minimization/maximization algorithm aims at finding the best (optimal) mapping function f:FeatureSpace→TargetSpace that maps the data to its targets based on the objective function J(w). The classification algorithms fall into Euclidean and Riemannian manifold based on how they interpret EEG feature space.

#### 2.7.1. Euclidean Space Methods

Euclidean space $R^{n}$ is the space of all *n*-dimensional real number vectors. Most of the classification algorithms work in this space. One of such algorithms is Decision Trees (DT) [81]. DTs creates a tree structure where each node f(x) (shown in Table 2) is a piece-wise function that outputs a child based on a feature $x_{i}$ and threshold *c*. Both the feature $x_{i}$ and the threshold *c* are determined by maximising (i.e., greedy algorithm) an objective function (e.g., gain impurity or information gain). This process is then repeated for each child output. If an output child does not improve the objective function, the node f(x) outputs a class ∈{1,−1} instead.

Linear discriminant analysis (LDA) [82] is an algorithm that creates a projection vector w that maximises the distance between classes $S_{B}$ and minimizes the variance within a class $S_{W}$$\left(J_{LDA}\left(w\right)=\max_{w\in R^{n}}\frac{w^{T}S_{B}w}{w^{T}S_{W}w}\right)$. This is done by finding a generalized eigenvector of $S_{B}w=\lambda S_{W}w$. The classification is achieved by finding a threshold *c* that separates both classes, such as, if the dot product is below the threshold *c*, it belongs to class 1; otherwise it belongs to class 2. Duda et al. [83] described extension of LDA for multi-class problem.

The support vector machine (SVM) is another classification algorithm that works in the Euclidean space [82]. We later discuss the extension of this algorithm into the Riemannian manifold. SVM works by projecting the data points ${\left\{x_{i}\right\}}_{i=1}^{M}$ onto a hyperplane H${\left\{\phi \left(x_{i}\right)\right\}}_{i=1}^{M}$. A plane in the hyperplane H is then created by solving the objective function (shown in Table 2) subject to $\alpha_{i}\geq 0$ and $\sum_{i}\alpha_{i}y_{i}=0$ using quadratic programming where $<,{>}_{H}$ is the dot product in hyperplane H. This plane is then used to distinguish between classes $f_{SVM}\left(x\right)=sgn\left(b+\sum_{i}y_{i}\alpha_{i}k\left(x,x_{i}\right)\right)$, where $k\left(x,x_{i}\right)=<\phi \left(x_{i}\right),\phi \left(x_{j}\right){>}_{H}$ is the hyperplane kernel. Different kernels exist for hyperplanes, such as the linear kernel $k\left(x,x_{i}\right)=x^{T}x_{i}$, the polynomial kernel $k\left(x,x_{i}\right)={\left(x^{T}x_{i}+c\right)}^{d}$, where *c* is a constant, and the exponential kernel $k\left(x,x_{i}\right)=\exp\left(-\gamma ∥x-x_{i}{∥}^{2}\right)$.

While DT, LDA and SVM have limited approximation abilities, multilayer perceptron (MLP) has no limits, as it is a universal approximate function. MLP $f\left(x\right)=\sum_{i}w_{i}^{\left(2\right)}\psi_{i}^{\left(1\right)}$$\left(\sum_{j}w_{j}^{\left(1\right)}x_{j}+b\right)$, as the name suggests, is a multilayer algorithms with each layer containing perceptrons that can fire ψ(.). The layers are connected by weights *w* that are trained using a minimization algorithm, such as stochastic gradient descent (SGD) or Adam algorithm. A convolutional neural network (CNN) is an extension to MLP. It extends the MLP algorithm by adding a convolution and pooling layers. In the convolution layer, the high-level information is extracted by using a matrix kernel that is applied to each part of the data matrix. While in the pooling layer, it extracts dominant features and decreases the computational power that is required to process the data by finding the maximum or average of the sub-matrices.

#### 2.7.2. Riemannian Space Methods

A Riemannian manifold is created when the EEG data are taken and converted into sample covariance matrices (SCM). This Riemannian manifold differs for the Euclidean space. For example, a metric for measuring distances between two points in the Riemannian manifold is not equivalent to its Euclidean counterpart. The minimum distance to Riemannian mean (MDRM) is the most popular classification algorithm in the Riemannian manifold [34]. MDRM is the extension of the Euclidean classification algorithm in the Riemannian manifold. This algorithm take in the data in the form of sample covariance matrices (SCM) and then calculates the Riemannian mean $\sigma \left(P_{1},\cdots ,P_{m}\right)\;=\;{arg min}_{P\in P(n)}\sum_{i=1}^{m}\delta_{R}^{2}\left(P,P_{i}\right)$ for each class using it to label data where $\delta_{R}\left(P_{1},P_{2}\right)=$$∥\mathrm{Log}\left(P_{1}^{-1}P_{2}\right){∥}_{F}={\left[\sum_{i=1}^{n}\log^{2}\lambda_{i}\right]}^{1/2}$ is the Riemannian distance. The Riemannian mean equation could be thought of as its objective functions J(P), while the algorithm that is used to find it could be conceptualised as a minimisation algorithm. MDRM has the following mapping function:

$$\begin{array}{c} f_{MDRM}\left(P_{m+1}\right)=\underset{j\in \{1,2,\cdots \}}{arg min}\delta_{R}\left(P_{m+1},P_{\Omega_{j}}\right) \end{array}$$

where $P_{\Omega_{j}}$ is the mean of class *j*. Similarly, Riemannian SVM (R-SVM) [34], is the natural extension of SVM algorithm into the Riemannian manifold. It uses the tangent space of a reference matrix $C_{\mathrm{ref}}$ as its hyper plane. This results in the following kernel:

$$\begin{array}{cc} k_{R}\left(\mathrm{vect}\left(C_{i}\right),\mathrm{vect}\left(C_{j}\right)\right);C_{\mathrm{ref}}) & =<\phi \left(C_{i}\right),\phi \left(C_{j}\right){>}_{C_{\mathrm{ref}}} \end{array}$$

where $\mathrm{vect}\left(C\right)=\left[C_{1,1};\sqrt{2}C_{1,2};C_{2,2};\sqrt{2}C_{1,3};\sqrt{2}C_{2,3};C_{3,3};\ldots ;C_{E,E}\right]$ is the vectorized form of a symmetric matrix, $\phi \left(C\right)={\mathrm{Log}}_{C_{\mathrm{ref}}}\left(C\right)$ is the map from the Riemannian manifold to the tangent space of $C_{\mathrm{ref}}$, and $<A,B{>}_{C}=\mathrm{tr}\left(AC^{-1}BC^{-1}\right)$ is the scalar product in the tangent space of $C_{\mathrm{ref}}$.

### 2.8. Performance Evaluation

The general architecture of motor-imagery based brain–computer interface is well understood, yet numerous novel MI based interfaces and strategies are proposed to enhance the performance of MI-BCI. Thus, performance evaluation metrics play an important role in quantifying diverse MI strategies. Accuracy is the most widely used performance evaluation, which measures the performance of algorithm in terms of correctly predicting target class trials. Accuracy metrics are mostly employed where the number of trials for all classes are equal and there is no bias towards a particular target class [84]. In the case of unbalanced (unequal number of trials) classes, Cohen’s kappa coefficient is employed [85]. The Kappa coefficient equates an observed accuracy with respect to an expected accuracy (random chance). If kappa coefficient is 0, it means that there is no correlation with the target class and predicted class, where, as kappa coefficient, 1 denotes perfect classification. If the MI classification is biased towards one class, then the confusion matrix (CM) is an important tool to quantify the performance of the system. Table 3 illustrates the confusion matrix for a multi-class problem. Metrics, like sensitivity and specificity, can be obtained from CM to identify the percentages of correctly classified trials from each MI class.

MI-BCI can be interpreted as a communication channel between user and machine, thus the information transfer rate (ITR) of each trial can be calculated in order to measure the bit-rate of the system. ITR can be obtained through CM (based on Accuracy) according to Wolpaw et al.’s [86] method as well as based on the performance and distribution of each MI classes [87]. The metrics discussed above are summarized in Table 4 and applicable for both synchronized and self-placed (asynchronized) as well as multi-class MI-BCIs. As a BCI can be defined as an encoder-decoder system where the user encodes information in EEG signals and the BCI decodes it into commands. The above metrics evaluate how well the BCI decode user’s MI task into commands, but it does not quantify how well the user modulates EEG patterns with MI tasks [88]. Therefore, there is room for improving performance metrics that measure user MI skills or a user’s encoding capability.

Lotte and Jeunet [88] have proposed stability and distinctiveness metrics to address some of the limitations mentioned above. Stability metrics measure how a stable MI EEG pattern is produced by a user. It is done by measuring the average distance between each MI task trial covariance matrix and mean covariance matrix for this MI task (left/right etc.). Distinct metrics measure the distinctiveness between MI task EEG patterns. Mathematically, distinct metrics is defined as the ratio of the between class variance to the within class variance. Stability and distinct metrics are both defined in the Riemannian manifold, as described in Table 4.

## 3. Key Issues in MI Based BCI

MI based BCI still face multiple issues for it to be commercially usable. A usable MI based BCI should be plug and play, self paced, highly responsive, and consistent, so that that everybody can use it. This could be achieved by solving the following challenges:

### 3.1. Enhancement of MI-BCI Performance

A high performance MI-based BCI is important, as it increases the responsiveness of the device and prevents user frustration, hence improving the users experience. Improving the performance could be achieved by improving its pre-processing stage, channel selection stage, feature selection stage, dimensionality reduction stage, or a combination of them.

#### 3.1.1. Enhancement of MI-BCI Performance Using Preprocessing

Recent enhancements in the pre-processing step have revolved around two aspects: enhancing the incoming signal or enhancing the filtering of the signal. The former can be achieved by reconstructing the signal [89,90], enhancing the spatial resolution [91], or adding artificial noise [92]. In Casals et al. [89], they reconstructed corrupted EEG channels by using a tensor completion algorithm. The tensor completion algorithm applied a mask to this corrupted data in order to estimate it from observed EEG data. They found that this reconstructed the data of the corrupted channels and improved the classification performance in MI-BCI, whereas Gaur et al. [90] used multivariate empirical mode decomposition (MEMD) to decompose the EEG signal into a set of intrinsic mode functions (IMFs). Based on a median frequency measure, a set of IMFs is selected to reconstruct EEG signals. The CSP features are extracted from the reconstructed EEG signal for classification. One can enhance the spatial resolution of the EEG signal by using local activities estimation (LAE) method [91]. The LAE method estimates the recorded value of an EEG channel based on the weighted sum of local values of all EEG channels. The weights that are assigned to each channel for a weighted sum are based on the distance between channels. Similarly, enhancing the filtering of the signal can be achieved by automated filter (subject specific) tuning based on optimization algorithm like particle swarm optimization (PSO), artificial bee colony (ABC), and genetic algorithm (GA) [93]. Kim et al. [94] and Sun et al. [95] both proposed filters that are aimed to remove artifacts. Kim et al. [94] removed ocular artifacts by using an adaptive filtering algorithm that was based on ICA. Sun et al. [95] removed EOG artifacts by a contralateral channel normalization model that aims at extracting EOG artifacts from the EEG signal while retaining MI-related neural potential through finding the weights of EOG artifact interference with the EEG recordings. The Hijorth parameter was then extracted from the enhanced EEG signal for classification. In contrast to the above methods, Sampanna and Mitaim [92] have used the PSO algorithm to search for the optimal Gaussian noise intensity to be added in signals. This helps in achieving higher accuracy when compared to a conventionally filtered EEG signal. The Signal that is reliable at run time is very important for online evaluation of MI-BCI. To address this, Sagha et al. [96] proposed a method that quantifies electrode reliability at run time. They proposed two metrics that are based on Mahalanobis distance and information theory to detect anomalous behaviour of EEG electrodes.

#### 3.1.2. Enhancement of MI-BCI Performance Using Channel Selection

Channel selection can both remove redundant and non-task relevant channels [97] and reduce power consumption of the device [98]. Removing channels can improve performance by reducing the search space [97], while reducing the power consumption can increase the longevity of a battery-based device [98]. Yang et al. [99] selected an optimal number of channels and time segments to extract features based on Fisher’s discriminant analysis. They used the F score to measure discrimination power of time domain features obtained from different channels and different time segments. Jing et al. [100] selected high quality trials (free from artifacts) to find an optimal channel for a subject based on the “maximum projection on primary electrodes”. These channels are used to calculate ICA filters for MI-BCI classification pipeline. This method has shown good improvement in classification accuracy even in session to session and subject to subject transfer MI-BCI scenarios. Park et al. [101] applied particle swarn optimization algorithm to find subject specific optimal number of electrodes. These electrodes’ EEG data is further used for classification. Jin et al. [102] selected electrodes that contain more correlated information. To do this, they applied Z-score normalization to EEG signals from different channels, and then computed pearson’s cofficients to measure the similarity between every pair of electrodes. From selected channels, RCSP features are extracted for SVM model based classification. This significantly improves the accuracy compared to traditional methods. Yu et al. [103] used Fly optimization algorithm (FOA) to select the best channel for subject and then extracted CSP features from these channels for the classification. They also compared FOA performance with GA and PSO. Ramakrishnan and Satyanarayana [98] used a large (64) and small (19) number of channels in data acquisition for training and testing phase, respectively. They calculated inverse Karhunen Loeve Tranform (KLT) matrix from training trials. This inverse KLT matrix is used to reconstruct all the missing channels in the testing phase. Masood et al. [104] employed various flavors of the CSP algorithm [31] to obtain the spatial filter weights of each electrode. Based on maximal values of spatial pattern coefficients, electrodes are selected to compute features for MI-CSP classification.

#### 3.1.3. Enhancement of MI-BCI Performance Using Feature Selection

Similar to channel selection, feature selection improves the performance by finding the most optimal features. Similarly, Yang et al. [105], in their study, decomposed EEG signals from C3,Cz, and C4 channels into a series of overlapping time-frequency areas. They achieved this by cutting the filtered signals from filter bank of width 4 Hz and step 1 Hz (e.g., 8–12,9–13,...26–30) into multiple overlapping time segments. They used an *F-score* to select optimal time-frequency areas to extract features for MI-BCI classification. Rajan and Devassy [106] used a boosting approach that improved the classification by a combination of feature vectors. Baboukani et al. [107] used an Ant Colony Optimization technique to select a subset of features for SVM based classification of MI-BCI. Wang et al. [108] divided all of the electrodes in several sensor groups. From these sensor groups, CSP features are extracted to calculate EDRs. These EDRs are fused together, based on information fusion to obtain discriminate features for ensemble classification. Liu et al. [109] proposed a feature selection method that is based on the firefly algorithm and learning automata. These selected features are used to classify by a spectral regression discriminant analysis (SRDA) classifier. Kumar et al. [110] used the mutual information technique to extract suitable features from CSP features from filter banks. Samanta et al. [111] used an auto encoder-based deep feature extraction technique to extract meaningful features from images of a brain connectivity matrix. The brain connectivity matrix is constructed based on mutual correlation between different electrodes.

#### 3.1.4. Enhancement of MI-BCI Performance Using Dimensionality Reduction

Xie et al. [112] learned low dimensional embedding on the Riemannian manifold based on prior information of EEG channels. Where, as, She et al. [113] extracted IMFs from EEG signals, and then employed Kernel spectral regression to reduce the dimension of IMFs. In doing so, they constructed a nearest neighbour graph to model the IMFs intrinsic structure. Özdenizci and Erdoğmuş [114] proposed the information theory based linear and non-linear feature transformation approach to select optimal feature for multi-class MI-EEG BCI system. Pei et al. [71] used stacked auto-encoders on spectral features to reduce the dimension and achieve high accuracy in a multi class asynchronous MI-BCI system. Razzak et al. [115] applied sparse PCA to reduce the dimensionality of features for SVM based classification. Horev et al. [55] extended the PCA to SPD manifold space, such that it preserved more variance in data while mapping SPD matrices to a lower dimension. Harandi et al. [116] proposed an algorithm that maintains the SPD matrices geometry while mapping it in a lower dimension. This is done by preserving the local structure’s distance with respect to the local mean. In addition to it, this mapping minimizes the geodesic distance in samples that belong to the same class as well as maximizes the geodesic distance between samples belonging to a different class. Davoudi et al. [79] adapted Harandi’s geometry preserving the dimensionality reduction technique in an unsupervised manner. Similarly, Tanaka et al. [80] proposed graph fourier transform for reducing dimensionality of SPD matrices through Tangent space mapping. This method has shown improvement in the performance for a small training dataset.

#### 3.1.5. Enhancement of MI-BCI Performance with Combination of All

Li et al. [117] used the TPCT imaging method to fix the electrode positions and assigned time-frequency feature values to each pixel in the MI-EEG image. This way promotes feature fusion from the time, space, and frequency domains, respectively. These high dimensional images are fed to the modified VGG16 network [118]. Wang et al. [119] extracted a subset of channels from the motor imagery region. From these extracted channels, a subject-specific time window and frequency band is obtained to extract CSP features for classification. Sadiq et al. [120] manually selected the channels from the sensory motor cortex area of the brain. The EEG signal from these selected channels is decomposed into ten IMFs using adaptive empirical wavelet transform. The most sensitive mode out of ten is selected based on PSD and the Hilbert transform (HT) method extracts the instantaneous amplitude (IA) and instantaneous frequency (IF) from each channel. The statistical features are extracted from IF and IA components for classification. Selim et al. [121] used the bio-inspired algorithm (attractor metagene (AM)) to select the optimal time interval and CSP features for classification. Furthermore, they used the Bat optimization algorithm (BA)) to optimize SVM parameters to enhance the classifier’s performance. Athif and Ren [122] proposed the wave CSP technique that used wavelet transform and CSP filtering technique to enhance the signal to noise ratio of the EEG signal and to obtain key features for classification. Li et al. [123] optimized the spatial filter by employing Fisher’s ration in objective function. This not only avoids using regularization parameters but also selects optimal features for classification. Li et al. [124] designed a spectral component CSP algorithm that utilized ICA to extract relevant motor information from EEG amplitude features that were obtained from CSP. Liu et al. [125] proposed an adaptive boosting algorithm that selects the most suitable EEG channels and frequency band for the CSP algorithm.

### 3.2. Reduce or Zero Calibration Time

Every day, a BCI user is required to go through a calibration phase for him/her to use BCI. This can be inconvenient, annoying, and frustrating. This section describes an on-going research solution to reduce the calibration phase or completely remove it. There are three categories of solutions: subject specific methods, transfer learning methods, and subject independent methods.

#### 3.2.1. Subject-Specific Methods

Subject-specific methods for the reduction of calibration time mostly aim at more efficiently extracting features (i.e., with a small amount of training data). This can be achieved by the particle swarm optimization based learning strategy to find optimal parameters for spiking neural model (SNM) (deep learning model) [126]. This method automatically adjusts the parameters, removes the need for manual tuning, and increases the efficiency of SNM. However, this requires very subject-specific optimization of the parameters for best results [127]. Whereas, Zhao et al. [128] proposed the use of a framework that transforms EEG signals into three-dimensional space to preserve the temporal and spatial distribution of EEG signal and uses multi-branch 3D convolutional neural network to take advantage of temporal and spatial features in EEG signal. They showed that this approach significantly improves the accuracy under a small training dataset. Another approach of reducing calibration time is by a subject specific modification of the CSP algorithm. For example, Park and Chung [129] improved CSP by electing the CSP features from good local channels, rather than all channels. They selected good local channels that are based on the variance ratio dispersion score (VRDS) and inter-class feature distance (ICFD). Furthermore, they extended this approach in Filter Bank CSP by selecting good local channels for each frequency band, whereas Ma et al. [130] optimized SVM classifier’s kernel and penalty parameters through a particle swarm optimization algorithm to obtain optimal CSP features. Furthermore, Costa et al. [131] proposed an adaptive CSP algorithm to overcome the limitation of CSP in short calibration sessions. They iteratively update the coefficients of the CSP filters while using a recursive least squares (RLS) approach. This algorithm can be enhanced based on right channel selection and training free BCI system by modifying the algorithm with unsupervised techniques. Kee et al. [25] proposed Renyi entropy as a new alternative feature extraction method for small sample setting MI-BCI. Their method outperforms conventional CSP and regularized CSP design in small training datasets. Lotte and Guan [31] proposed Weighted Tikhonov Regularization for the CSP objective function that gives different penalties for different channels based on their degree of usefulness to classify a given mental state. They also extended the conventional CSP method for a small sample setting in [132] by penalizing the CSP objective function through prior information of EEG channels. Prior information of EEG channels was also used by Singh et al. [133] to obtain a smooth spatial filter in order to reduce the dimension of covariance matrices of trials under a small training set. They used MDRM for the classification of covariance matrices. This approach has shown higher performance under a high dimensional small sample setting.

#### 3.2.2. Transfer Learning Methods

An investigation on inter-session and inter-subject variabilities in multi-class MI-based BCI revealed the feasibility of developing calibration-free BCIs in subjects sharing common sensorimotor dynamics [134]. Transfer learning methods have been developed based on this concept of using other subjects/sessions. Transfer learning methods aim to use other subjects data either to increase the amount of data that the classifier can be trained on or to regularize (prevent overfitting) the algorithm. The former can be seen in He and Wu [135], Hossain et al. [136], and Dai et al. [137]. He and Wu [135] used Euclidean-space alignment (EA) on the top of CSP to enable transfer learning from other subjects. EA projects all subjects into a similar distribution while using the Euclidean mean. Hossain et al. [136] extended FBCSP by adding selective informative instance transfer learning (SIITAL). The SIITAL trains the FBCSP with both source and target subjects by iteratively training the model and selecting the most relevant samples of the source subjects based on that model. Dai et al. [137] proposed unified cross-domain learning framework that uses the FBRCSP method [138] to extract the features from source and target subjects. This is achieved by ensemble classifiers that are trained on misclassified samples and contribute to the overall model based on their classification accuracy, while the latter can be seen in Azab et al. [139], Singh et al. [140,141], Park and Lee [138], and Jiao et al. [142]. Azab et al. [139] proposed a logistic regression-based transfer learning approach that assigns different weights to a previously recorded session or source subject in order to represent similarities between these sessions/subjects features distribution and the new subject features distribution. Based on Kullback–Leibler divergence (KL) metrics, similar source/session feature space to target subject is chosen to obtain subject-specific common spatial patterns features for classification. Singh et al. [140,141] proposed a framework that takes advantage of both Euclidean and Riemannian approaches. They used a Euclidean subject to subject transfer approach to obtain optimized spatial filter for the target subject and employed Riemannian geometry-based classification to take advantage of the geometry of covariance matrices. Park and Lee [138] extended the FBCSP with regularization. They obtained an optimized spatial filter for each frequency band using information from other subjects’ trials. The CSP features from each frequency band are obtained and, finally, based on mutual information most discriminate CSP features are selected for classification. Jiao et al. [142] proposed Sparse Group Representation Model for reducing the calibration time. In their work, they constructed a composite dictionary matrix with training samples from source subjects and target subject. A sparse representation-based model is then used to estimate the most compact representation of a target subject samples for classification by explicitly exploiting within-group sparse and group-wise sparse constraints in the dictionary matrix. The former has the advantage of being applicable to all the trained subjects over the latter.

#### 3.2.3. Subject Independent Methods

Subject-independent methods aim to eliminate the calibration stage, allowing for the user to plug and play the BCI device. One way of achieving this is by projecting all different subjects/sessions’ data into a unified space. Rodrigues et al. [143] proposed the Riemannian Procrustes Analysis as a projection based method. It transforms subject-specific data into a unified space by applying a sequence of geometrical transformations on their SCMs. These geometrical transformations aim to match the distribution of all subjects in high-dimensional space. These geometrically transformed SCMs are then fed to the MDRM classification model to discriminate the MI tasks. However, this method still requires the creation of the geometrical transformations that are based on the targets’ session; thus, it is not entirely calibration-free, but it paves the way for fully subject independent MI-BCIs. Another way of achieving subject-independence is to create a universal map that can take in any subject data and output the command. Zhu et al. [144] proposed a deep learning framework for creating a universal neural network, called separate channel CNN (SCCN). SCCN contains three blocks: the CSP block, Encoder block, and recognition block. The CSP block was used to extract the temporal features from each channel. The encoder block then encodes those extracted features, followed by a concatenation of the encoded features and feeding them into the recognition block for classification. Joadder et al. [145] also proposed a universal MI-BCI map that extracts sub-band energy, fractal dimension, Log Variance, and Root Mean Square (RMS) features from spatial filtered EEG signal (CSP) for Linear Discriminant Analysis (LDA) classification model. They evaluated their design on a different time window after cue, different frequency band and different number of EEG channels and obtained good performance as compared to existing subject-dependent methods. Although both Zhu et al. [144] and Joadder et al. [145] classifiers are subject-independent, the CSP extracted features are not. Zhao et al. [146] hypothesized that there exists a universal CSP that is subject-independent. They used a multi-subject multi-subset approach where they took each subject in the training data and randomly picked samples to create multiple subsets and calculated a CSP on each subset. This was followed by a fitness evaluation-based distance between these CSP vectors (density and distance between highly dense vectors). They also proposed a semi-supervised approach as a classifier; however, unlike the universal CSP, it required unlabelled target data. In the same vein, Kwon et al. [147] followed the same universal CSP concept. Unlike Zhao et al. [146], they only trained one CSP on all of the available source subject’s data and, since they had a larger dataset, they assumed that it would find the universal CSP. Mutual information and CNN was then used for a complete subject-independent algorithm.

### 3.3. BCI Illiteracy

BCI illiteracy subject is defined as the subject who cannot achieve a classification accuracy higher than 70% [11,148,149,150,151,152,153]. BCI illiteracy indicates that the user is unable to generate required oscillatory pattern during MI task. This leads to poor performance of MI-BCI. Some of the researchers focus on predicting whether a user falls under BCI illiterate category or not. This can help us to design a better algorithm for decoding MI or designing better training protocol to improve user skills. For instance, Ahn et al. [154] demonstrated that self-assessed motor imagery accuracy prediction has a positive correlation with actual performance. This can be valuable information to find BCI inefficiency in the user. While, Shu et al. [149], in their work, proposed two physiological variables, which is, laterality index (LI) and cortical activation strength (CAS), to predict MI-BCI performance prior to clinical BCI usage. Their proposed predictors exhibited a linear correlation with BCI performance, whereas Darvishi et al. [155] proposed a simple reaction time (SRT) as the BCI performance predictor. SRT is a metric that reflects the time that is required for a subject to respond to a defined stimulus. Their results indicate that SRT is correlated with BCI performance and BCI performance can be enhanced if the feedback interval is updated in accordance with the subject’s SRT. In the same vein, Müller et al. [156] has theoretically shown that adaptation that is too fast may confuse the user, while an adaptation that is too slow might not be able to track EEG variabilities due to learning. They created an online co-adaptation BCI system by ever-changing feedback according to the user and the system’s learning. In the same vein, the co-adaptive approach to address BCI illiteracy has also been proposed by Acqualagna et al. [150]. Their paradigm was composed of two algorithms: a pre-trained subject independent classifier based on simple features, and a supervised subject optimized algorithm that can be modified to run in an unsupervised setting based manner. The approach of Acqualagna et al. is based on the classification of users put forth by Vidaurre et al. [157]. Vidaurre et al. [157], in their study, classified users in three categories: for category I users (Cat I), the classifier can be successfully trained and they gain good BCI control in the online feedback session. For Category II users (Cat II), the classifier can be successfully trained; however, good performance cannot be achieved in the feedback phase. For Category III users (Cat III), successful training of the classifier is not achieved. In the same vein, Lee et al. [158] found that that a universal BCI illiterate user does not exist (i.e., all of the participants were able to control at least one type of BCI system). Their study paves way to design a BCI system based on user’s skill.

Another way of addressing BCI illiteracy problem is to design novel solutions that can improve performance, even in the case of BCI illiterate user. Similarly, Zhang et al. [153] addressed BCI illiteracy through a combination of CSP and brain network features. They constructed a task-related brain network by calculating the coherence between EEG channels, the graph-based analysis showed that the node degree and clustering coefficient have intensity differences between left and right-hand motor imagery. Their work suggests that there is a need to explore more feature extraction methods to address the BCI illiteracy problem. Furthermore, Yao et al. [148] proposed a hybrid BCI system to address the BCI inefficiency that is based on somatosensory attentional (SA) and motor imagery (MI) modalities. SA and MI are generated by attentional concentration intention (at some focused body part) and mentally simulating the kinesthetic movement, respectively. SA and MI are reflected through EEG signals at the somatosensory and motor cortices, respectively. In their work, they demonstrate that the combination of SA and MI would provide distinctive features to improve performance and increase the number of commands in a BCI system. In the same vein, Sannelli et al. [159] created an ensemble of adaptive spatial filters to increase BCI performance for BCI inefficient users. External factors can also improve BCI accuracy. For instance, Vidaurre et al. [160] proposed assistive peripheral electrical stimulation to modulate activity in the sensorimotor cortex. It is proposed that this will elicit short-term and long-term improvements in sensorimotor function, thus improving BCI illiteracy among users.

### 3.4. Asynchronised MI-BCI

MI-based BCI is usually trained in a synchronous manner, which is, there exists a sequence of instructions (or cue) that a user follows to produce an ERD/ERS phenomenon. However, in a real-world application, user want to execute control signal at his own will rather than waiting for cue. Therefore, there has been an increasing interest in creating an asynchronous MI. That is, MI-based BCI can detect that the user has an intention to undertake motor imagery, and then classifies MI task. This is done by splitting the incoming data into segments with overlapping periods. Each segment represents a potential MI command. One way of determining whether this potential MI command is an actual MI command is to build a classifier for that purpose. For example, the study of Yu et al. [161] presents the self-paced operation of a brain–computer interface (BCI), which can be voluntarily used to control a movement of a car (starting the engine, turning right, turning left, moving forward, moving backward, and stopping the engine). The system involved two classifiers: control intention classifier (CIC) and left/right classifier (LRC). The CIC is implemented in the first phase to identify the user intention being “idle” or “MI task-related”. If an MI task-related is identified, a second phase follows the first phase by classifying it. Similarly, both Cheng et al. [162] and Antelis et al. [163] proposed a deep learning method that is trained to distinguish between resting state, transition state, and execution state. However, Cheng proposed a convolutional neural network, followed by a fully connected network (CNN-FC), while Antelis proposed Dendrite morphological neural networks (DMNN). Another approach is to let the subject achieve a set number of consistent right/left classification within a set period for an action to be taken, thus confirming the command and avoiding randomness [164], both adding a classifier and classifying multiple times, adds computational time and complexity to the system, with the latter also adding the time required for classification. Sun et al. [165] suggested a method that avoids these constraints by using a threshold on an existing classifier that separates idle from MI task-related. He et al. [166] proposed a similar approach for continuous application, such as mouse movement. This is achieved through moving the object (in this case a mouse) by the confidence level of the classifier. The threshold-based method of addressing this challenge requires defining a threshold that could be difficult and user-dependent. This brings us to the last methodology of addressing this challenge, which is, by adding an idle class into the classifier [167,168,169,170]. All of the above-motioned methods, except the method proposed by Yousefi et al. [170], use a target-oriented paradigm where the user is asked to perform a task and the algorithm is evaluated based on the user’s ability to achieve that task. However, Yousefi et al. [170] tested their algorithm by giving the user a specified time interval to perform any task the user desired and after the time has passed, the user provides feedback as to whether the algorithm responded to his commands. In conclusion, all of the algorithms can run asynchronously, given that they have a reasonable run time.

### 3.5. Increase Number of Commands

More diverse and complex applications, like spellers etcetera, can be developed with high ITR and increased number of classes in MI-BCI. Traditionally, MI-BCI was designed as binary class (left and right) problem. The first way to extend MI-BCI into multi-class is by employing a hybrid approach during which the MI paradigm is complemented with other mental strategies. For example, Yu et al. [171] proposed a hybrid asynchronous brain–computer interface that is based on sequential motor imagery (MI) and P300 potential to execute eleven control functions for wheelchairs. The second way to achieve multi-class MI-BCI is algorithmically. For example, the traditional CSP algorithm is extended to recognize four MI tasks [172]. In the similar manner, Wentrup and Buss [173] proposed information theoretic feature extraction frameworks for CSP algorithm by extending it for multiclass MI-BCI system. In the same vein, Christensen et al. [174] extended FBCSPs for five class MI-BCI system. Similarly, Razzak et al. [175] proposed a novel multiclass support matrix machine to handle multiclass MI imagery tasks. Likewise, Barachant et al. [176] presented a new classification method based on Riemannian geometry that uses covariance matrices to classify multi-class BCI. Faiz and Hamadani [177] controlled humonoid robotic hand gentures through five class online MI BCI while using a commercial EEG headset. They user AR and CSP feature extractions and PCA to reduce the dimension of AR features. Finally, CSP and AR features are concatenated and trained by a SVM classifier to achieve multi-class recognition.

### 3.6. Adaptive BCI

The consistency of the accuracy of the classifier during long sessions is one of the issues still being worked in EEG based MI-BCI. This is because EEG is a non-stationary signal that get impacted over time as well as when there is change in recording environment and state of mind (e.g., fatigue, attention, motivation, emotion, etc). Adaptive methods have been proposed to address this challenge. For instance, Aliakbaryhosseinabadi et al. [178] demonstrated that it is possible to detect a user’s attention diversion during a MI task, whereas Dagaev et al. [179] extracted the target state (LH, RH) from background state (environment, emotional, and cognitive condition, etc.). This was achieved by asking subjects in the training stage to open and close their eyes during the trials. These instructions act as the two different background conditions. The methods that detect cause of change in user signals other than the MI task could pave the way for adaptive MI-BCI by giving both the user real-time neurofeedback and giving the adaptive algorithm additional information to work with while decoding MI task.

Another way to address this challenge is to modify the training protocol or extracting more information during it. Mondini et al. [180] and Schwarz et al. [181] both modified the training protocol. By creating an adaptive training protocol, Mondini et al. [180] fulfiled three tasks: (a) adapt the training session based on the subject’s ability, which is, make the training short and restart the training from the beginning with different motor imagery strategy if the system performance is lower than a certain threshold; (b) present training cue (left/right) in a biased manner that is present left cue more often manner if the left imagery performance is low when compared to the right; and, (c) keep challenging the performance of the user by only giving feedback if it exceeds an adaptive threshold. Schwarz et al. [181] proposed a co-adaptive online learning BCI model that uses the concept of semi-supervised retraining. The Schwarz model uses a few initial supervised calibration trials per MI tasks and then performs recurrent retraining by using artificially generated labels. This ensures feedback to the user after a very short training and engages the user in mutual learning with the system. Information gathered during training protocol, such as command delivery time (CDT) and the probability of the next action, could be used to address this challenge. Saeedi et al. used CDT [182] to provide a system that delivers adaptive assistance, which is, if the current trial is long, then the system will slow down to give enough time to the user to execute the MI tasks. Their study suggests that the brain pattern is different for short, long and time-out commands. They were able to differentiate between command type using only one second before the trial started, while Perdikis et al. [183] proposed using the probability of next action to adapt the classifier. Specifically, they implemented an online speller based on the BrainTree MI text-entry system that uses probabilistic contextual information to adapt an LDA classifier. The final method observed in the literature to address this challenge was to create an adaptive classifier. Faller et al. [184] proposed an online adaptive MI-BCI that auto-calibrates. Their system in regular interval not only discriminates features for classifier retraining, but also learns to reject outliers. Their system starts to provide feedback after minimal training and keeps improving by learning subject-specific parameters on the run. Raza et al. [185] proposed an unsupervised adaptive ensemble learning algorithm that tackles non-stationary based co-variate shifts between two BCI sessions. This algorithm paves the way for online adaption to variabilities between BCI sessions. In the same vein, Rong et al. [186] proposed an online method that handles the statistical difference between sessions. They used an adaptive fuzzy inference system.

### 3.7. Online MI-BCI

After an adaptive BCI, the BCI mode is one key factor that determines MI based system’s usability and efficacy. MI-BCI systems are operated in offline or online mode through cue-based paradigms, where self-placed (asynchronous) are mostly online systems. Mostly, the literature proposed improvements in offline mode of MI-BCI systems; very few test their proposed algorithms in the online environment. In online BCI studies, Sharghian et al. [187] proposed MI-EEG, which uses sparse representation-based classification (SRC). Their approach obtains an online dictionary learning scheme from the extracted band power from a spatial-filtered signal. This dictionary leads to reconstruction of sparse signal for classification. In the same vein, Zhang et al. [188] proposed an incremental linear discriminant analysis algorithm that extract AR features from preferable incoming data. Their method paved way for fully auto-calibrating an online MI-BCI system. Similarly, Yu et al. [167] proposed an asynchronous MI BCI system to control wheelchair navigation. Perez [189] extended the fuzzy logic framework for adaptive online MI-BCI system and evaluated it through the realistic navigation of a bipedal robot. Ang and Guan [190] introduced an adaptive strategy that continuously computes the subject-specific model during an online phase. Abdalsalam et al. [191] controlled the screen cursor through a four class MI-BCI system. Their results suggest that online feedback increases ERDs over the mu (8–10 Hz) and upper beta (18–24 Hz) band, which results in a higher cursor control success rate. Many studies have demonstrated the efficiency of virtual reality (VR) and gaming environment in a online BCI [192]. Achanccaray et al. [193] in the same vein, verified that virtual reality based online feedback has positive effects on the subject. It has been observed that motor cortex increases its activation level (in alpha and beta band) due to an immersive VR experience. This is very helpful in supporting upper limb rehabilitation of post-stroke patients. Similarly, Alchalabi and Faubert [194] used VR based neurofeedback in the Online MI-BCI session. Cubero et al. [195] proposed an online system that is based on an endless running game that runs on three class MI-BCI. They used graphic representation of EEG signals for multi-resolution analysis to take advantage of spatial dimension, along with temporal and spectral dimensions.

### 3.8. Training Protocol

Similar to other normal user skills, BCI control is also a skill that can be learned and improved with proper training. A typical BCI training protocol is a combination of user instructions, cues on screen to modulate the user’s neural activity in a specific manner, and, lastly, a feedback mechanism that represents confidence of the classifier in recognition of the mental task to user. Unfortunately, standard training protocol does not satisfy the psychology of human learning; usually being boring and very long. Meng and He [196] studied the effect of MI training on users. They found out that, with a few hours of MI training, there is change in electrophysiological properties. Their study suggested design engaging training protocol and multiple training sessions, rather than a long training session for low BCI performers. In the same vein, Kim et al. [197] proposed a self placed training protocol, in which the user performs MI task continuously without an inter-stimulus-interval. During each trial, the user has to imagine a single MI task (e.g., RH for 60 s). The results of this protocol showed that it reduces the calibration time when compared to conventional MI training protocol. Jeunet et al. [198] surveyed the cognitive and psychological factors that are related to MI-BCI and categorized these factors into three categories (a) user-technology relationship, (b) attention, and (c) spatial abilities. Their work is very useful for designing a new training protocol that takes advantage of these factors. Furthermore, in another study, Jeunet et al. [11] found that spatial ability plays an important role in BCI performance of a subject. They suggested having pre-training sessions to explore spatial ability for BCI training.

Many studies proposed new training strategies that use other mental strategies to compliment MI training (kinesthetic imagination of limbs). For instance, Zhang et al. [199] proposed a new BCI training paradigm that combines conventional MI training protocol with covert verb reading. This improves the performance of MI-BCI and paves the way for utilizing semantic processing with motor imagery. Along the same lines, Wang et al. [200] proposed a hybrid MI-paradigm that uses speech imagery with motor imagery. In this paradigm, the user repeatedly and silently reads move (left/right) cues during imagination. Standard training protocols are fixed that are not tailored made to user’s need and experience. With respect to this, Wang et al. [201] proposed MI training with visual-haptic neurofeedback. Their findings validate that their approach improves cortical activations at the sensorimotor area, thus leading to an improvement in BCI performance. Liburkina et al. [202] proposed a MI training protocol that gives cue to perform and feedback to the user through vibration. Along the same lines, Pillette et al. [203] designed an intelligent tutoring system that provides support during MI training and enhance user experience/performance on MI-BCI system. Skola et al. [204] proposed a virtual reality-based MI-BCI training that uses a virtual avatar to provide feedback. Their training helps in maintaining high levels of attention and motivation.Furthermore, their proposed method improves the BCI skills of first time users.

## 4. Conclusions

In this paper, we have provided an extensive review of methodologies for designing an MI-BCI system. In doing so, we have created a generic framework and mapped literature related to different components (data acquisition, MI training, preprocessing, feature extraction, channel and feature selection, classification, and performance metrics) in it. This will help in visualizing gaps to be filled by future studies in order to further improve BCI usability.

Despite many outstanding developments in MI-BCI research, some critical issues still need to be resolved. Mostly, studies are on synchronized MI-BCI in offline mode. There is a need to have more studies on online BCI. Typically, researchers use performance evaluation metrics, as per their convenience. It would be better to have general BCI standards that can be widely adhered by researchers. Our literature survey found that enhancing the performance is still a critical issue even after two decades of research. Due to availability of high computational resources, present studies employ methods based on deep learning and Riemannian geometry more than traditional machine learning methods. With current advancement in algorithms, future research should concentrate more on eliminating or reducing long calibration in MI-BCI. Future studies should focus on more diverse BCI applications that can be developed with increased number of commands. Our review shows that BCI illiteracy is a critical issue that can be addressed either by using better training protocol that suit users’ requirements or through smart algorithms. Finally, EEG is a non-stationary signal that changes over time as user’s state of mind changes. This causes inconsistency in the BCI classifier’s performance; thus, it is important to make progress in development of adaptive methods in order to address this challenge in an online settings.

## Figures and Tables

**Table 1:** This table provides a summary of the feature extraction methods.

A Summary of Feature Extraction Methods		
Temporal methods	Statistical Features [19,20]	$Mean\left(\bar{x}\right)=\frac{1}{T}\sum_{t=1}^{T}\left|x_{t}\right|$,
		$Std.Dev\left(\sigma \right)=\sqrt{\frac{1}{T}\sum_{t=1}^{T}{\left(x_{t}-\bar{x}\right)}^{2}}$
		$Variance\left(x\right)=\frac{1}{T}\sum_{t=1}^{T}{\left(x_{t}-\bar{x}\right)}^{2}$
		$skewness=\frac{\frac{1}{T}\sum_{t=1}^{T}{\left(x_{t}-\bar{x}\right)}^{3}}{\sigma^{3}}$
		$kurtosis=\frac{\frac{1}{T}\sum_{t=1}^{T}{\left(x_{t}-\bar{x}\right)}^{3}}{\sigma^{4}}$
	Hijorth features [21]	$Activity=\frac{\sum_{t=1}^{T}{\left(x_{t}-\bar{x}\right)}^{2}}{T}$
		$Mobility=\frac{Var(\hat{x_{t}})}{Var(x_{t})}$
		$Complexity=\frac{Mobility(\hat{x_{t}})}{Mobility(x_{t})}$
	RMS [20]	$RMS_{t}=\sqrt{\frac{1}{N}\sum_{i-1}^{N}x_{i}^{2}}$
	IEEG [20]	$IEEG_{t}=\sum_{i=1}^{N}|x_{I}$
	Fractal Dimension [22]	$D=\frac{\log(L/a)}{\log(d/a)}$
	Autoregressive modeling [21]	$x_{t}=\sum_{i=1}^{p}a_{i}x_{t-i}+\epsilon_{t}$ where {a for i = 1,…, p} are AR model coefficients and p is the model order
	Peak-Valley modeling [23,24]	Cosine angles, Euclidean distance between neighbouring peak and valley points
	Entropy [25,26]	$S=-\sum_{i=1}^{N}p_{i}\ln p_{i}$
	Quaternion modeling [27]	$Mean\left(\mu \right)=\frac{\sum (q_{mod})}{N}$ $Variance\left(\sigma \right)=\frac{{\left(\sum {\left(q_{mod}\right)}^{2}-\mu \right)}^{2}+\sum {\left(q_{mod}\right)}^{2}}{2N}$ $Contrast\left(con\right)=\frac{\sum {\left(q_{mod}\right)}^{2}}{N}$ $Homogeneity\left(H\right)=\sum \frac{\left(1\right)}{1+{\left(q_{mod}\right)}^{2}}$ $ClusterShade\left(cs\right)=\sum {\left(q_{mod}-\mu \right)}^{3}$ $Clusterprominence\left(cp\right)=\sum {\left(q_{mod}-\mu \right)}^{4}$
Spectral methods	Band power [19]	$F\left(s\right)=\sum_{n=0}^{N-1}x_{n}e^{\frac{-2\pi }{N}sn},s=0,1,\ldots ,N-1$ $Pow\left(s\right)=\int_{f_{l}ow}^{f_{h}igh}F{\left(s\right)}^{2}ds$
	Spectral Entropy [26]	$SH=-\sum_{f1}^{f2}\hat{P}\left(f\right)\log\left(\hat{P}\left(f\right)\right)$ $\hat{P}\left(f\right)=P\left(f\right)/\sum_{f1}^{f2}P\left(f\right)$, P(f) is PSD of signal
	Spectral statistical Features [19]	Mean Peak Frequency, Mean Power, Variance of Central Frequency etc.
Time-frequency Methods	STFT [28]	$S\left(m,k\right)=\sum_{n=0}^{N-1}s\left(n+mN\right)w\left(n\right)e^{-j\frac{2\pi }{N}nk}$
	Wavelet transform [29]	$\psi_{s,\tau }\left(t\right)=\frac{1}{\sqrt{s}}\psi \left(\frac{t-\tau }{s}\right)$
	EMD [30]	$x\left(t\right)=\sum_{i=1}^{n}c_{i}\left(t\right)+r_{n}\left(t\right)$
Spatial Methods	CSP [31]	$J\left(w\right)=\frac{w^{T}C_{1}w}{w^{T}C_{2}w}$
	BSS [32,33]	x(t)=As(t) $s^{\prime }\left(t\right)=Bx\left(t\right)$ Approaches like ICA, CCD estimate $s^{\prime }\left(t\right)$
Spatio-temporal methods	Sample covariance matrices [34]	$C_{i}=\frac{X_{i}X_{i}^{T}}{tr(X_{i}X_{i}^{T})}$ Where $C_{i}$ is covariance matrix of single trial

**Table 2:** This table provides a summary of the classification methods described in the Section 2.7.

	Mapping Function	Objective Function	Min/Max Algorithm
DT	$f\left(x\right)=\left\{\begin{array}{cc} child_{1} & x_{i}\leq c\\ child_{2} & otherwise \end{array}\right.$	Gain impurity, information gain	greedy algorithm
LDA	$f\left(x\right)=\left\{\begin{array}{cc} 1 & w^{T}x<c\\ -1 & otherwise \end{array}\right.$	$J\left(w\right)=\max_{w\in R^{n}}\frac{w^{T}S_{B}w}{w^{T}S_{W}w}$	Eigen value solver
SVM	$f\left(x\right)=sgn(b+\sum_{i}y_{i}\alpha_{i}k\left(x,x_{i}\right))$	$\max_{\alpha \in R^{m}}\left(\sum_{i}\alpha_{i}-\frac{1}{2}\sum_{i,j}\alpha_{i}\alpha_{j}y_{i}y_{j}k\left(x_{i},x_{j}\right)\right)$	Quadratic Programming
R-SVM			
MLP	$f\left(x\right)=\sum_{i}w_{i}\psi_{i}\left(.\right)$	MSE, Cross entropy, Hinge	SGD, Adam
CNN	f(x)=conv+pool+MLP		
MDRM	$f\left(P\right)={arg min}_{j\in \{1,2,\cdots \}}\delta_{R}\left(P,P_{\Omega_{j}}\right)$	$J\left(P_{\Omega }\right)={arg min}_{P_{\Omega }\in P\left(n\right)}\sum_{i}\delta_{R}^{2}\left(P_{\Omega },P_{i}\right)$	Averaging approaches

**Table 3:** Multi class Confusion matrix.

		Prediction			
		${\mathrm{Class}}_{1}$	${\mathrm{Class}}_{2}$	${\mathrm{Class}}_{k}$	${\mathrm{Class}}_{n}$
Target	$Class_{1}$	$D_{11}$	$D_{12}$	$D_{1k}$	$D_{1n}$
	$Class_{2}$	$D_{21}$	$D_{22}$	$D_{2k}$	$D_{2n}$
	$Class_{k}$	$D_{k1}$	$D_{k2}$	$D_{kk}$	$D_{kn}$
	−	−	−	−	−
	$Class_{n}$	$D_{n1}$	$D_{n2}$	$D_{n3}$	$D_{nn}$

**Table 4:** Summary of all the Metrics.

	Metrics	Two Class	Multi Class (N-Class)
BCI decoding capabilty	Accuracy	$\frac{D_{11}+D_{22}}{N_{all}}$	$\frac{\sum_{i=1}^{N}D_{ii}}{N_{all}}$, where $N_{all}=\sum_{i,j=1}^{N}D_{ij}$
	Kappa	$\frac{Accuracy-expected\;accuracy}{1-expected\;accuracy}$, $expected\;accuracy\left(A_{e}\right)=\frac{\sum_{i=1}N}{D_{i:}D_{:i}}$	
	sensitivity	$\frac{D_{22}}{D_{21}+D_{22}}$	$\frac{D_{kk}}{N_{k}}$, where $N_{k}=\sum_{i=1}^{N}D_{k,i}$
	ITR${}_{Wolpaw}$	${\mathrm{ITR}}_{Wolpaw}=\log_{2}N+Acc.\log_{2}\left(Acc\right)+\left(1-Acc\right)\log_{2}\left(\frac{1-Acc}{N-1}\right)$	
User encoding capability	Stability	$\frac{1}{1+\sigma_{C^{A}}}$	
	Distinct	$\frac{\sum_{1}^{N}\delta_{r}\left(\bar{C^{A_{i}}},\bar{\bar{C^{A}}}\right)}{\sum_{1}^{N}\sigma_{C^{A_{i}}}}$	

## Data Availability

Not applicable.
